# CD4^+^CD25^+^LAG3^+^ T Cells With a Feature of Th17 Cells Associated With Systemic Lupus Erythematosus Disease Activity

**DOI:** 10.3389/fimmu.2019.01619

**Published:** 2019-07-12

**Authors:** Rika Kato, Shuji Sumitomo, Yumi Tsuchida, Haruka Tsuchiya, Shinichiro Nakachi, Keiichi Sakurai, Norio Hanata, Yasuo Nagafuchi, Kanae Kubo, Shoko Tateishi, Hiroko Kanda, Tomohisa Okamura, Kazuhiko Yamamoto, Keishi Fujio

**Affiliations:** ^1^Department of Allergy and Rheumatology, Graduate School of Medicine, The University of Tokyo, Tokyo, Japan; ^2^Department of Immunotherapy Management, Graduate School of Medicine, The University of Tokyo, Tokyo, Japan; ^3^Max Planck-The University of Tokyo Center for Integrative Inflammology, The University of Tokyo, Tokyo, Japan; ^4^Center for Integrative Medical Sciences, The Institute of Physical and Chemical Research, Yokohama, Japan

**Keywords:** systematic lupus erythematosus, LAG3, regulatory T cells, Th17, SLEDAI

## Abstract

Systemic lupus erythematosus (SLE) is an autoimmune disease that involves multiple immune cell subsets. We analyzed immune cell subsets in human peripheral blood mononuclear cells (PBMC) in order to identify the cells that are significantly associated with SLE disease activity and treatment. The frequencies of various subsets of CD4^+^ T cells, B cells, monocytes and NK cells in PBMC were assessed in 30 healthy controls (HC), 30 rheumatoid arthritis (RA) patients and 26 SLE patients using flow cytometry. The correlations between subset frequencies in SLE and clinical traits including Systemic Lupus Erythematosus Disease Activity Index (SLEDAI) were examined. Changes in subset frequencies after the treatment in SLE patients were investigated. We focused on CD25^+^LAG3^+^ T cells and investigated their characteristics, including cytokine secretion, mRNA expression and suppression capacity. We assessed correlations between CD25^+^LAG3^+^ T cells and SLEDAI by Spearman's rank correlation coefficient. CD25^+^LAG3^+^ T cells were significantly increased in SLE whereas there were few in RA and HC groups. CD25^+^LAG3^+^ T cell frequencies were significantly correlated with SLEDAI and were increased in patients with a high SLEDAI score (> 10). CD25^+^LAG3^+^ T cells produced both IL-17 and FOXP3, expressed mRNA of both *FOXP3* and *RORC* and lacked suppressive capacity. CD25^+^LAG3^+^ T cells were associated with disease activity of SLE. CD25^+^LAG3^+^ T cells had features of both CD25^+^FOXP3^+^ regulatory T cells (CD25^+^ Treg) and Th17. CD25^+^LAG3^+^ T cells could be associated with the inflammatory pathophysiology of SLE.

## Introduction

Systemic lupus erythematosus (SLE) is an autoimmune disease that is characterized by loss of tolerance, production of autoantibodies, immune complex deposition, and end organ damage. Multiple immune cell subsets are involved in the pathophysiology of SLE. SLE appears to be induced by persistent apoptotic debris (1) that primes neutrophils' NETosis (2), induces plasmacytoid dendritic cells to produce type I interferons that reduce B cell tolerance (3) and decreases regulatory T cell (Treg) function (4).

There are many reports demonstrating changes in the frequencies of immune cell subsets in peripheral blood mononuclear cells (PBMCs) of SLE patients. Whereas, naïve CD4^+^ T cells (5) decreased in frequency in SLE, activated effector memory T cells (6), Th1 (7), Th17 (8), and follicular helper T cells (Tfh) (9) all increased. Naïve B cells decreased, and plasmablasts and plasma cells increased in SLE (10, 11). On the other hand, in SLE patients, the reported changes in the frequencies of CD4^+^CD25^+^ regulatory T cells (CD25 Tregs) that express *FOXP3* (12, 13) are still controversial (14–16).

At present, it is not clear which PBMC subsets are significantly correlated with SLE disease activity. SLE pathology is reportedly associated with Th17 (8), FOXP3^+^Helios^+^ Treg (17, 18), and plasma cells (19). Based on available studies, we concluded that comprehensive analysis of immune cell subsets was necessary to directly compare the association between disease activity and individual immune cell subsets. The present analysis of human PBMC was standardized by using the gating and staining strategies recommended by the Human Immunological Project Consortium (HIPC) (20).

Here, we observed a correlation between the frequencies of specific cell subsets and clinical traits in SLE, and the effect of treatment on the frequencies of those cell subsets. We included an expression analysis of lymphocyte activation gene 3 (LAG3) in a CD4^+^ regulatory T cell analysis panel. LAG3 is a member of the immunoglobulin superfamily that strongly binds to MHC class II (21). LAG3-expressing cells were identified as IL-10-producing regulatory T cells (Tr1) in human PBMC (22), and CD4^+^LAG3^+^ T cells were usually negative for both CD25 and *FOXP3* expression. Human CD4^+^CD25^−^LAG3^+^ T cells (CD25^−^LAG3^+^ T cell) were detected in both PBMC (23, 24) and tonsils (25) that produced high amounts of IL-10, expressed low levels of *FOXP3*, and suppressed antibody production of B cells.

## Materials and Methods

### Human Samples and Clinical Data

We recruited 26 SLE patients, 30 rheumatoid arthritis (RA) patients and 30 self-reported screened healthy controls (HC). Individuals under 20 years of age or with active infection were excluded. SLE patients fulfilled the 1982 American College of Rheumatology criteria for SLE or the 2012 SLICC Classification criteria for SLE. RA patients fulfilled the 1987 revised criteria of the American College of Rheumatology or the 2010 ACR/EULAR classification criteria. The following clinical data were collected for both SLE and RA patients: disease duration, white blood cell count (WBC) (normal range is 3,300–8,600 cells/μL), and total lymphocyte count calculated by multiplying the WBC concentration by the percentage of lymphocytes in a complete blood cell count. The following clinical data were collected only in SLE: SLE disease activity index (SLEDAI) (26), total complement activity (CH_50_) (normal range is 31.8–48.7 U/mL), and anti-double stranded DNA (dsDNA) antibody titer measured by fluorescence-enzyme immunoassay (cut off value is 10 IU/mL). All clinical investigations conformed to the Declaration of Helsinki principles and were approved by the ethics committee of the University of Tokyo (No. 10154 and G3582). Peripheral blood and clinical data were collected after getting written informed consent in accordance with our ethical review board. Peripheral blood was collected once from each donor, except for four SLE patients whose samples were taken multiple times during the course of treatment. In the case of one SLE sample, the frequencies of cell subsets (except for activated CD25^+^ Treg, CD25^+^ Treg, CD25^−^LAG3^+^ T cell, and CD25^+^LAG3^+^ T cells) were excluded because of a change of gating strategy.

### Flow Cytometric Analysis

**Figure 1:**
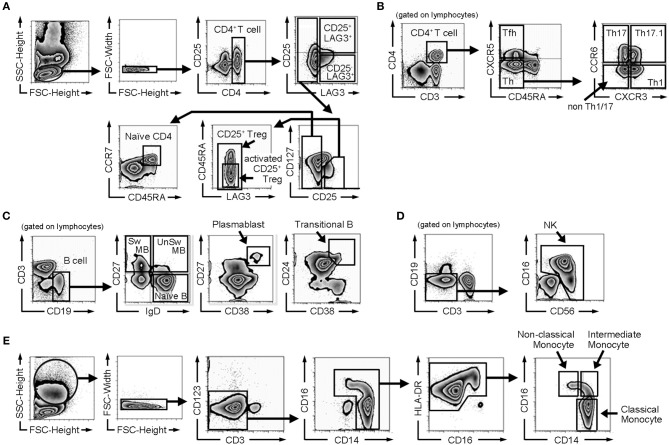
Gating strategy of flow cytometric analysis. **(A)** Gating strategy for naïve CD4^+^ T cells, CD25^+^ regulatory T cells (CD25^+^ Tregs), activated CD25^+^ regulatory T cells (activated CD25^+^ Tregs), CD25^−^LAG3 T cells, and CD25^+^LAG3^+^ T cells. **(B)** Gating strategy for follicular helper T cells (Tfh), helper T cells (Th), Th1, Th17, Th1/17, and non Th1/17. **(C)** Gating strategy for B cell subsets (naïve B cells, switched memory B cells (Sw MB), unswitched memory B cells (UnSw MB), plasmablasts and transitional B cells). **(D)** Gating strategy for NK cells. **(E)** Gating strategy for monocyte subsets (classical monocytes, intermediate monocytes and non-classical monocytes).

PBMCs were isolated from whole blood by Ficoll-Paque Plus (GE Healthcare) gradient separation. Fc Receptor Binding Inhibitor (eBioscience) was added to the isolated PBMCs. They were stained with the fluorescent-labeled antibodies for 20 min. The following antibodies were purchased from BioLegend: CD3-PerCP/Cy5.5 (UCHT1), CD3-PE/Cy7 (UCHT1), CD27-FITC (O323), CD38-PE/Cy7 (HIT2), CD19-APC/Cy7 (HIB19), CD45RA-APC/Cy7 (HI100), CD16-PerCP/Cy5.5 (3G8), CD56-APC/Cy7 (HCD56), CCR7-PerCP/Cy5.5 (G043H7), CD14-FITC (M5E2), and CD25-BV421 (BC96). The following antibodies were purchased from BD: CD24-PE (ML5), IgD-BV421 (IA6-2), CXCR5-AF488 (RF8B2), CCR6-PE (11A9), CXCR3-BV421 (1C6/CXCR3), CD4-V500 (RPA-T4) and CD19-V500 (HIB19). CD25-PE/Cy7 (BC96), HLADR-PE (L243), and CD127-PE/Cy7 (eBioRDR5) were purchased from eBioscience, and LAG3-PE (FAB2319P) was purchased from R&D. We classified CD4^+^ T cells, B cells, natural killer (NK) cells and monocytes based on the Human Immunology Project classification (20). Cell subset definitions of surface markers are shown in Table 1, and the gating strategy is shown in Figure 1. Flow cytometric analyses were performed by 8-color MoFlo XDP (Beckman Coulter). Flow cytometric data were analyzed using FlowJo software (Tree Star).

**Table 1:** Cell subset definition.

**Subset name (Abbreviation)**	**Definition of surface marker**
CD4^+^ T cell	CD4^+^
Naïve CD4^+^ T cell	CD4^+^CD25^−^LAG3^−^CCR7^+^CD45RA^+^
Activated CD25^+^ regulatory T cell (activated CD25^+^ Treg)	CD4^+^CD25^+^LAG3^−^CD127^low^CD45RA^−^
CD25^+^ regulatory T cell (CD25^+^ Treg)	CD4^+^CD25^+^LAG3^−^CD127^low^
CD25^−^LAG3^+^ T cell	CD4^+^CD25^−^LAG3^+^
CD25^+^LAG3^+^ T cell	CD4^+^CD25^+^LAG3^+^
T helper type 1 cell (Th1)	CD4^+^CD45RA^−^CXCR5^−^CXCR3^+^CCR6^−^
T helper type 17 cell (Th17)	CD4^+^CD45RA^−^CXCR5^−^CXCR3^−^CCR6^+^
T helper type 1/17 cell (Th1/17)	CD4^+^CD45RA^−^CXCR5^−^CXCR3^+^CCR6^+^
Non T helper type 1/17 cell (non Th1/17)	CD4^+^CD45RA^−^CXCR5^−^CXCR3^−^CCR6^−^
Follicular helper T cell (Tfh)	CD4^+^CD45RA^−^CXCR5^+^
B cell	CD3^−^CD19^+^
Naïve B cell	CD3^−^CD19^+^IgD^+^CD27^−^
Unswitched memory B cell (UnSw MB)	CD3^−^CD19^+^IgD^+^CD27^+^
Switched memory B cell (Sw MB)	CD3^−^CD19^+^IgD^−^CD27^+^
Plasmablast (PB)	CD3^−^CD19^+^IgD^−^CD27^high^CD38^high^
Transitional B cell (Trans B)	CD3^−^CD19^+^CD24^high^CD38^high^
Monocyte	CD3^−^CD19^−^CD56^−^HLA-DR^+^CD14^+^
Classical monocyte (Class Mono)	CD3^−^CD19^−^CD56^−^HLA-DR^+^CD14^high^CD16^−^
Intermediate monocyte (Int Mono)	CD3^−^CD19^−^CD56^−^HLA-DR^+^CD14^high^CD16^+^
Non-classical monocyte (Non-class Mono)	CD3^−^CD19^−^CD56^−^HLA-DR^+^CD14^dim^CD16^+^
Natural killer cell (NK)	CD3^−^CD19^−^CD14^−^CD56^+^

### RNA Isolation, cDNA Synthesis, and Quantitative Real-Time PCR

Naïve CD4^+^ T cells, activated CD25^+^ Tregs, CD25^−^LAG3^+^ T cells, CD25^+^LAG3^+^ T cells were sorted and stimulated for 72 h on flat-bottom 96-well microplates pre-coated with anti-CD3ε monoclonal antibody (mAb) (10 μg/mL) and anti-human CD28 mAb (5 μg/mL). The culture medium was RPMI 1640 medium supplemented with 10% fetal bovine serum (FBS), 2 mM L-glutamine, 100 U/mL penicillin, 100 mg/mL streptomycin, 50 mM 2-ME (all purchased from Life Technologies) and recombinant human IL-2 (100 IU/mL, R&D). After staining with 7-AAD Viability Staining Solution (BioLegend), 7-AAD-negative cells were sorted.

For *FOXP3* expression analysis, sorted Naïve CD4^+^ T cells, activated CD25^+^ Tregs, CD25^−^LAG3^+^ T cells, CD25^+^LAG3^+^ T cells were analyzed. For *RORC* expression analysis, sorted Naïve CD4^+^ T cells, activated CD25^+^ Tregs, CD25^−^LAG3^+^ T cells, CD25^+^LAG3^+^ T cells stimulated for 72 h with anti-CD3ε monoclonal antibody (mAb) (10 μg/mL) and anti-human CD28 mAb (5 μg/mL) were analyzed. Total RNA was extracted using the RNeasy Micro Kit (QIAGEN) and then reverse-transcribed to cDNA with random primers (Invitrogen) and Superscript III (Invitrogen), according to the manufacturer's protocol. To determine the cellular expression of each gene, quantitative real-time PCR analysis was performed using CFX connect (Bio-Rad). The PCR mixture consisted of 10 μL SYBR Green Master Mix (QIAGEN), 15 pM forward and reverse primers, and the cDNA samples in a total volume of 20 μL. We calculated the quantitative PCR data with the D threshold cycle method, and relative RNA abundance was determined based on control *GAPDH* abundance. The real-time PCR primer pairs were as follows: human *FOXP3* sense, 5′-GAAACAGCACATTCCCAGAGTTC-3′ and antisense, 5′-ATGGCCCAGCGGATGAG-3′; human *RORC* sense, 5′-CAGTCATGAGAACACAAATTGAAGTG-3′ and antisense, 5′-CAGGTGATAACCCCGTAGTGGAT-3′; human *GAPDH* sense, 5′-GAAGGTGAAGGTCGGAGTC-3′ and antisense, 5′-GAAGATGGTGATGGGATTTC-3′.

### Intracellular Staining Analysis

Naïve CD4^+^ T cells, activated CD25^+^ Tregs, CD25^−^LAG3^+^ T cells, CD25^+^LAG3^+^ T cells, and CD4^+^CD25^−^LAG3^−^CD45RA^−^ T cells (Memory CD4^+^ T cells) were sorted and stimulated for 72 h with anti-CD3ε mAb (10 μg/mL) and anti-human CD28 mAb (5 μg/mL) in the presence of recombinant human IL-2 (100 IU/mL). Twelve hours prior to cytokine production analysis, phorbol 12-myristate 13-acetate (PMA) (25 ng/mL), ionomycin (1 μg/mL) and protein transport inhibitor GolgiStop (BD) were added. After staining with 7-AAD, intracellular staining was performed using Cytofix/Cytoperm Fixation/Permeabilization Kit (BD) following the manufacturer's instructions. For cytokine production analysis, IFN-γ-FITC (4S.B3, eBioscience), IL-4-APC (8D4-8, BioLegend), IL-17A-APC (eBio64DEC17, eBioscience) or IL-10-Alexa Fluor 660 (JES3-9D7, eBioscience) antibodies were used. For staining Foxp3, Foxp3 Staining Buffer Set (eBioscience) and Foxp3-FITC (PCH101, eBioscience) antibody were used.

### T Cell Suppression Assay

CD4^+^ naïve T cells were purified by magnetic cell separation (MACS) using the Naïve CD4^+^ T Cell Isolation Kit II (Miltenyi Biotech), after which cells were labeled with 2 mM CFSE (Dojindo). CD3-negative cells were sorted by flow cytometry and used as antigen presenting cells (APCs) after 30 Gy irradiation. CFSE-labeled CD4^+^ naïve T cells (5 × 10^4^) and 1 × 10^5^ APCs were co-cultured with 5 x 10^4^ CD4^+^ naïve T cells, activated CD25^+^ Tregs or CD25^+^LAG3^+^ T cells on U-bottom 96-well plates that had been coated with anti-CD3ε mAb (10 μg/mL) and anti-human CD28 mAb (5 μg/mL) overnight. The decrease in CFSE intensity in viable cells was assessed 72 h later.

### Statistical Analysis

Data are presented as means ± standard deviation. To evaluate statistical differences between two unpaired groups, Mann-Whitney U test was used. To evaluate statistical differences between paired samples, paired Student's *t*-test was used. To compare three or more groups, Kruskal-Wallis test followed by Dunn's multiple comparison test or one-way ANOVA followed by Tukey's multiple comparisons test was used. To assess correlations, Spearman's rank correlation coefficient was used. To assess categorical data, Fisher's exact test was used. *P*-values <0.05 were considered statistically significant. Calculations were conducted using GraphPad Prism version 5.03 (GraphPad Software Inc.) and R version 3.2.3.

## Results

### Clinical Data

Clinical data describing 26 SLE patients, 30 RA patients and 30 HC donors are summarized in Table 2. The ages of SLE and HC were not significantly different, although the ages were significantly higher in RA. There was no significant difference in the sex ratio among SLE, RA, and HC. The WBC count was elevated in RA compared with HC, and the lymphocyte count was significantly lower in SLE compared with HC. In SLE, the average of total complement activity (CH_50_) was within normal range (31.8–48.7 U/mL), while anti-dsDNA antibody titer was higher than the cut off value (10 IU/mL). The average SLEDAI score was 9.6, and 5 patients (19.2%) had lupus nephritis, and 8 patients (30.8%) had neuropsychiatric SLE (NPSLE).

**Table 2:** Clinical data.

	**HC (*n* = 30)**	**RA (*n* = 30)**	**SLE (*n* = 26)**
Age	34.6 ± 8.0	54.5 ± 13.8^****^^a^ ^**^^b^	42.9 ± 15.3
Male/Female–n	7/23	8/22	2/24
SLEDAI–median (IQR)	–	–	8 (9.5)
Lupus nephritis–*n*, (%)	–	–	5 (19.2)
NPSLE–*n*, (%)	–	–	8 (30.8)
WBC (/μL)	5,266 ± 1,332	7,143 ± 2,171^**^^c^	6,108 ± 2,889
Lymphocyte (/μL)	1,859 ± 644	1,523 ± 576	1,284 ± 875 ^**^^d^
CH_50_ (U/mL)	–	–	36.2 ± 16.6
Anti-dsDNA antibody (U/mL)	–	–	87.9 ± 130.2

*Data are presented as mean ± SD, unless otherwise specified. For SLEDAI, median with interquartile range (IQR). HC, Healthy Control; RA, Rheumatoid Arthritis; SLE, Systemic Lupus Erythematosus; SLEDAI, SLE Disease Activity Index; NPSLE, Neuropsychiatric SLE; WBC, White Blood cell count; CH_50_, total complement activity; Anti-dsDNA antibody, Anti double stranded DNA antibody*.

** *0.001 < P < 0.01*,

**** *P < 0.0001, Categorical data were tested with Fisher's exact test. Continuous data were tested with Kruskal-Wallis test followed by Dunn's multiple comparison test*.

a *High compared with HC*,

b *high compared with SLE*,

c *high compared with HC*,

d *low compared with HC*.

### In SLE, Immature Cell Frequencies Decreased and Activated Cells Increased in Both T and B Cell Populations

**Figure 2:**
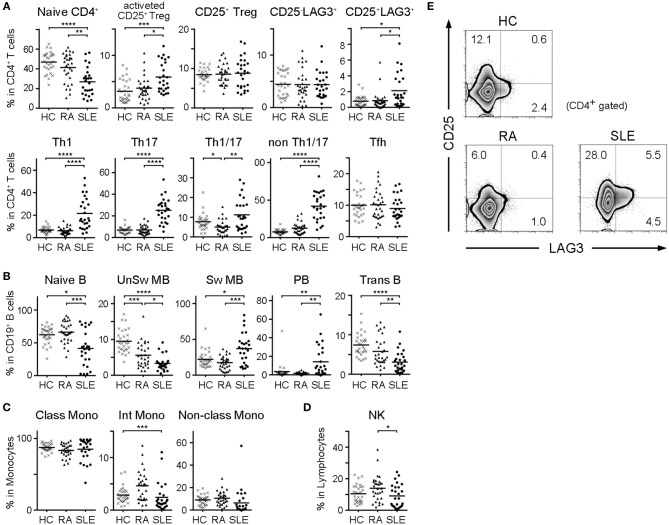
Frequencies of cell subsets in PBMC of HC, RA, and SLE. With regard to SLE samples taken multiple times from the same patient during treatment, the first sample taken before treatment was chosen. **(A)** Frequencies of CD4^+^ T cell subsets in CD4^+^ T cells. **(B)** Frequencies of B cell subsets in CD19^+^ B cells. **(C)** Frequencies of monocyte subsets in monocytes. **(D)** Frequencies of NK cells in lymphocytes. Small horizontal lines indicates the mean. ^*^P < 0.05; ^**^P < 0.01; ^***^P < 0.001; ^****^P < 0.0001. Kruskal-Wallis test followed by Dunn's multiple comparison test. **(E)** Representative flow cytometric findings showing CD25 and LAG3 expression in CD4^+^ gated PBMC of HC, RA, and SLE.

We compared subset frequencies of CD4^+^ T cells, B cells, monocytes and NK cells among HC, RA and SLE (Figures 2A–D). Compared to HC and RA, the frequency of naïve CD4^+^ T cells was significantly lower, and the frequencies of activated CD25^+^ Tregs, CD25^+^LAG3^+^ T cells, Th1, Th2 and Th17 were significantly higher in SLE (Figure 2A). With regard to B cell subsets, the frequencies of naïve B cells, unswitched memory B cells (UnSw MB) and transitional B cells (Trans B) were significantly lower in SLE compared to both HC and RA (Figure 2B). In contrast, the frequencies of switched memory B cells (Sw MB) and plasmablasts (PB) were significantly higher in SLE compared to HC and RA (Figure 2B). In both T and B cells, we commonly observed that naïve or inactive cells were decreased whereas activated and mature cells were increased in SLE. Representative flow cytometric findings shown in Figure 2E indicated that the frequency of CD25^+^LAG3^+^ T cells was higher in SLE compared with both HC and RA.

### The Frequency of CD25^+^LAG3^+^ T Cells Was High in Active SLE and Positively Correlated With SLEDAI

**Figure 3:**
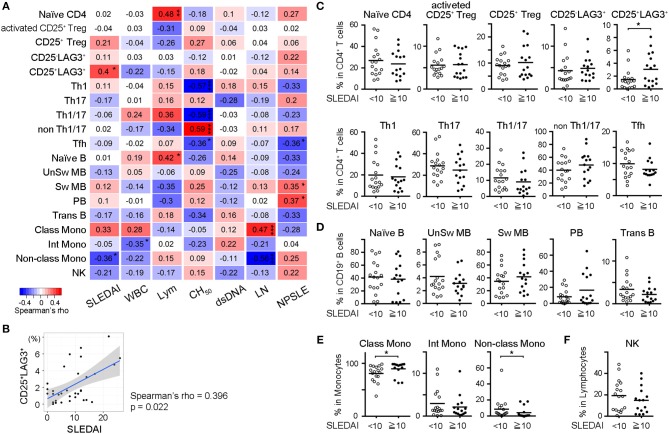
Frequencies of CD25^+^LAG3^+^ T cells correlated with high disease activity of SLE. **(A)** Correlogram showing the correlation between frequencies of cell subsets in SLE and clinical traits including SLEDAI, white blood cell count (WBC), lymphocyte count (Lym), total complement activity (CH_50_), anti-dsDNA antibody titer (dsDNA), history of lupus nephritis (LN), and history of neuropsychiatric SLE (NPSLE). Analyses were performed on all cases including samples taken multiple times during treatment [*n* = 33 (activated CD25^+^ Treg, CD25^+^ Treg, CD25^−^LAG3^+^, and CD25^+^LAG3^+^), *n* = 32 (other subsets)]. Numbers in the matrix show Spearman's rho. ^*^P < 0.05; ^**^P <0.01; ^***^P <0.001. Spearman's rank correlation coefficient. **(B)** Scatter plot of frequencies of CD25^+^LAG3^+^ cells vs. SLEDAI score (*n* = 33). **(C–F)** Frequencies of PBMC subsets in SLE were compared between patients with SLEDAI less than 10 (*n* = 17) and patients with SLEDAI >10 [*n* = 16 (activated CD25^+^ Treg, CD25^+^ Treg, CD25^−^LAG3^+^, and CD25^+^LAG3^+^), *n* = 15 (remaining subsets)]. ^*^P < 0.05; Mann-Whitney U test. **(C)** CD4^+^ T cell subsets, **(D)** B cell subsets, **(E)** Monocyte subset, and **(F)** NK cell.

In order to identify the subset that was best correlated with the clinical state of SLE patients, we calculated Spearman's rank correlation coefficient between frequencies of cell subsets and SLE clinical traits including SLEDAI, WBC count, lymphocyte count, total complement activity, anti-dsDNA antibody titer, history of lupus nephritis (LN) and history of neuropsychiatric SLE (NPSLE) (Figure 3A). Analyses were performed on all cases, including samples taken multiple times during treatment. The frequencies of CD25^+^LAG3^+^ T cells and CD14^dim^CD16^+^ monocytes (non-classical monocytes) significantly correlated with SLEDAI scores. A scatter plot of the frequencies of CD25^+^LAG3^+^ cells vs. SLEDAI scores showed a significantly positive correlation (Spearman's rho = 0.396, *P* = 0.022) (Figure 3B). We divided SLE patients into two groups according to the SLEDAI 10, and compared frequencies of cell subsets (Figures 3C–F). In active SLE, the frequencies of CD25^+^LAG3^+^T cells and CD14^+^CD16^−^ cells (classical monocytes) were significantly higher and the frequency of non-classical monocytes was significantly lower.

### CD25^+^LAG3^+^T Cells Decreased After Treatment

**Figure 4:**
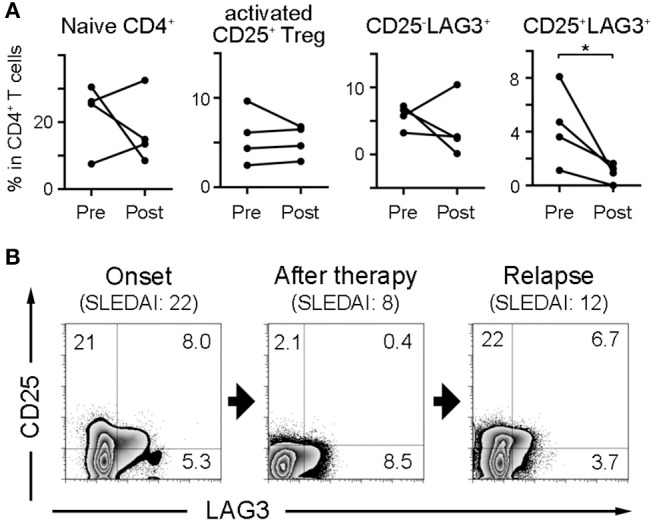
Frequencies of CD25^+^LAG3^+^ T cells decrease after SLE treatment. **(A)** Four SLE patients were sequentially analyzed before and after treatment. Frequencies of naïve CD4^+^ T cells and regulatory T cell subsets were compared between before (Pre) and after treatment (Post). ^*^P < 0.05; paired-T test. **(B)** Sequential flow cytometric findings showing CD25 and LAG3 expression in CD4^+^ gated PBMC of a patient with SLE. Analysis was performed at the onset with NPSLE (SLEDAI = 22), the resolution 2 months after treatment (SLEDAI = 8) and the relapse with new skin rash 10 months later (SLEDAI = 12).

Four SLE patients were sequentially analyzed before and after treatment, and the effect of treatment on the frequencies of naïve CD4^+^ T cells and regulatory T cell subsets was observed (Figure 4A). CD25^+^LAG3^+^T cells had a significant tendency to decrease after treatment. One SLE patient with NPSLE at the onset (SLEDAI = 22) resolved 2 months after treatment (SLEDAI = 8), and relapsed with new skin rash 10 months later (SLEDAI = 12). The frequency of CD25^+^LAG3^+^T cells decreased after treatment, but increased again coincident with SLE activity (Figure 4B).

### CD25^+^LAG3^+^ T Cells Have Features of Both CD25^+^ Treg and Th17

**Figure 5:**
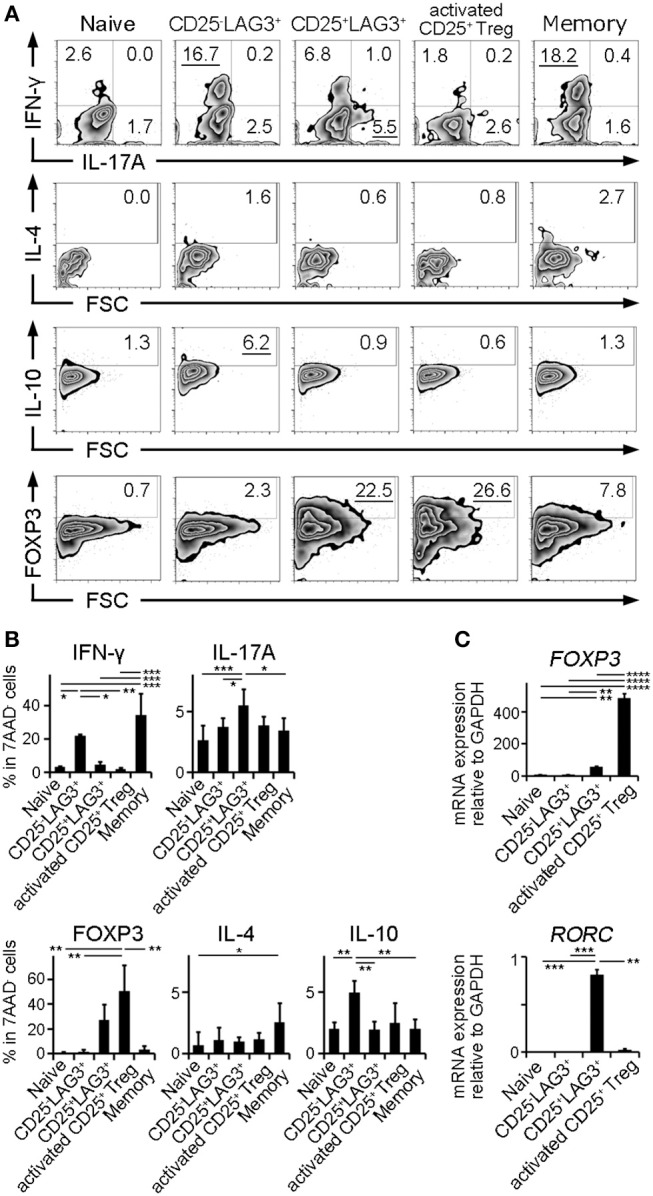
Cytokine and mRNA expression analysis. Naïve CD4^+^ T cells (Naïve), CD25^−^LAG3^+^ T cells (CD25^−^LAG3^+^), CD25^+^LAG3^+^ T cells (CD25^+^LAG3^+^), activated CD25^+^ Tregs and CD4^+^CD25^−^LAG3^−^CD45RA^−^ T cells (Memory) donated by HC were analyzed. **(A)** Representative findings of intracellular staining. CD4^+^ T cell subsets were separated by sorting and stimulated for 72 h with anti-CD3ε mAb (10 μg/mL) and anti-human CD28 mAb (5 μg/mL) in the presence of recombinant human IL-2 (100 IU/mL). **(B)** Summary of intracellular staining of CD4^+^ T cell subsets taken from three HC individuals. ^*^P < 0.05; ^**^P < 0.01; ^***^P < 0.001. One-way ANOVA followed by Tukey's multiple comparisons test. **(C)** Relative mRNA expression of *FOXP3* compared to *GAPDH* in sorted CD4^+^ T cell subsets (*n* = 3) without stimulation. Relative mRNA expression of *RORC* compared to *GAPDH* in sorted CD4^+^ T cell subsets (*n* = 3) stimulated for 72 h with anti-CD3ε mAb (10 μg/mL) and anti-human CD28 mAb (5 μg/mL) in the presence of recombinant human IL-2 (100 IU/mL). Cell subsets were ^**^P < 0.01; ^***^P < 0.001; ^****^P < 0.0001. One-way ANOVA followed by Tukey's multiple comparisons test.

In order to clarify the character of cell subsets, naïve CD4^+^ T cells, CD25^−^LAG3^+^ T cells, CD25^+^LAG3^+^ T cells, activated CD25^+^ Tregs and CD4^+^CD25^−^LAG3^−^CD45RA^−^ T cells (Memory CD4^+^ T cells) were sorted from the PBMC donated by HC. Intracellular staining was performed to evaluate the production of cytokines and the expression of FOXP3. Representative flow cytometric findings showed that CD25^−^LAG3^+^ T cells produced high amounts of both IFN-γ and IL-10, in agreement with an earlier reports (23, 24). CD25^+^LAG3^+^ T cells expressed high amounts of both IL-17A and FOXP3 (Figure 5A). Intracellular staining of three HC individuals showed that CD25^−^LAG3^+^ T cells and memory CD4^+^ T cells expressed high amounts of IFN-γ. Activated CD25^+^ Tregs expressed the highest amount of FOXP3, and CD25^+^LAG3^+^ T cells expressed the secondary highest amount of FOXP3. CD25^+^LAG3^+^ T cells expressed the higher amount of IL-17A compared with naïve, memory and CD25^−^LAG3^+^ T cells (Figure 5B). With regard to mRNA expression, activated CD25^+^ Treg expressed the highest amount of *FOXP3*. CD25^+^LAG3^+^T cells expressed a fairly small amount of *FOXP3*, and high amounts of *RORC* (Figure 5C).

### CD25^+^LAG3^+^ T Cells Did Not Show Suppressive Activity

**Figure 6:**
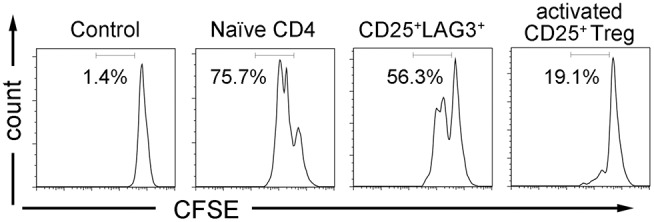
T cell suppression assay. CFSE labeled CD4^+^ naïve T cells were co-cultured with irradiated APCs and CD4^+^ T cell subsets (naïve CD4^+^ T cells, CD25^+^LAG3^+^ T cells and activated CD25^+^ Tregs) in the presence of anti-CD3ε mAb (10 μg/mL) and anti-human CD28 mAb (5 μg/mL). CFSE dilution of viable cells was assessed 72 h later. Flow cytometric data show results from one representative experiment out of three independent experiments. Percentages of proliferating cells are presented.

Activated CD25^+^ Treg and CD25^+^LAG3^+^ T cells were sorted, and we investigated their suppressive ability by co-culturing them with CFSE-labeled naïve CD4^+^ T cells and antigen presenting cells. While activated CD25^+^ Treg showed the expected suppressive activity, CD25^+^LAG3^+^ T cells did not show the evident suppressive activity as activated CD25^+^ Treg demonstrated (Figure 6).

## Discussion

We compared the frequencies of various immune cell subsets in PBMC collected from HC, RA and SLE groups. Our observations in SLE were consistent with previous reports showing decreases of naïve CD4^+^ T cells (5, 6) and naïve B cells (10) and an increase of memory B cells and plasmablasts (11, 19).

About markers of Treg, CD25 and *FOXP3* can be expressed by activated non-Tregs (27) without a stable regulatory phenotype (28). CD25 is a poor marker of Tregs in SLE because of the high number of non-Treg cells in the CD25^high^ population, therefore several markers had been proposed to evaluate Treg in SLE. GITR, which is expressed at high levels in activated T cells and Treg (29), was used to identify CD4^+^CD25^low/−^GITR^+^ cells that expand in SLE patients with inactive disease that exert a high inhibitory activity (30). Moreover, Helios, an Ikaros family member, was used to identify FOXP3^+^Helios^+^ cells as activated CD25^+^ Tregs that were increased in SLE (18). We defined CD4^+^CD25^+^CD127^low^CD45RA^−^ T cells as activated CD25^+^ Treg since this definition is widely accepted to enrich activated CD25^+^ Treg in human samples (31). We confirmed that the frequency of activated CD25^+^ Treg was significantly higher in SLE as shown earlier (32, 33). On the other hand, these subsets were not significantly correlated with SLEDAI.

We found that CD25^+^LAG3^+^ T cells was significantly increased in SLE. The frequency of CD25^+^LAG3^+^ T cells was significantly correlated with SLEDAI and decreased after treatment. These results implied that CD25^+^LAG3^+^ T cells might be involved in the pathophysiology of SLE. CD25^+^LAG3^+^ T cells expressed *FOXP3*, albeit less than activated CD25^+^ Tregs did, but they did not have apparent regulatory activity. We suggest that the expression of *FOXP3* in CD25^+^LAG3^+^ T cells resulted from T cell activation in SLE (34).

On the other hand, CD25^+^LAG3^+^ T cells expressed a high amount of *RORC* and IL-17 as well as *FOXP3*, a characteristic of IL-17-producing FOXP3^+^ T cells (35). Apart from the reciprocal relationship of Th17 and Treg cells, the plasticity between Th17 and Foxp3^+^CD25^+^ Treg has been observed (36). The *in vitro* conversion from Foxp3^+^CD25^+^Treg to Th17-like Tregs is dependent on IL-1β and on epigenetic modification (37). IL-17-secreting Tregs express RORγt (36) and were suppressive *in vitro* (38), but they rapidly lost their suppressive capacity upon strong activation in the presence of IL-1β and IL-6 (39). There also seems to exist mechanisms that allow the rapid shutdown of suppression and the induction of pro-inflammatory responses in Treg cells. Tumor infiltrating Tregs were rapidly converted into Th17 cells, down-regulated FOXP3 expression and lost their suppressive capacity (40).

About the issue of the switch between Th17 and Treg cells, it is still difficult to conclude the origin of CD25^+^LAG3^+^ T cells. There is the possibility that Th17 shifted to Treg (41). Considering the result of qPCR of unstimulated cells (Figure 5C), CD25^+^LAG3^+^ T cells might be a transient state of Th17 cells switching to the Treg phenotype. However, there are more reports that Treg shifted to IL-17-producing FOXP3^+^ T cells (35–37). Immunological milieu in SLE that both innate immunity and acquired immunity are activated might induce shift from Foxp3^+^CD25^+^ Treg to IL-17-producing FOXP3^+^ T cells. More confirmations should be performed.

There are several reports regarding the relevance of IL-17-producing FOXP3^+^ T cells in autoimmune diseases. IL-17-producing FOXP3^+^ T cells were highly enriched within the inflammatory environment of childhood arthritis, suggesting a role in the disease (42). IL-17-producing FOXP3^+^ T cells were recently associated with psoriasis (43) and systemic sclerosis (44). Recently, it was reported that IL-17-producing FOXP3^+^ T cells were associated with pathogenicity in a lupus model mouse in a RORγt-dependent manner (45). These cells potently suppress anti-inflammatory Th2 immunity in a RORγt-dependent manner, and they advocates these cells as novel players in SLE.

The reason why the expression of LAG3 in CD4^+^CD25^+^ T cells could be a marker of IL-17-producing FOXP3^+^ T cells remains to be solved. It was recently reported that environmental stimuli-induced intraepithelial lymphocytes (IELs) in the gut had a Th17-like profile and markedly upregulate LAG3 expression (46). They proliferated in response to gut-derived antigens and potentially prevent autoimmunity. Moreover, CCR9^+^ memory T cells in CSF of patients with secondary progressive multiple sclerosis expressed high level of LAG3 and RORγt. They produced high amount of IL-17A and presented a loss of regulatory function (47). FOXP3^+^RORγt^+^ Tregs were increased in PBMC from patients with pancreatic ductal adenocarcinoma, and they produced IL-17A and expressed high level of LAG3. They suppressed T cell immune responses, but enhanced inflammation (48). Thus, there are several reports of LAG3 expression on Th17 type cells. LAG3 is a surface marker that can be expressed by activation (49), therefore immunological milieu in SLE that induce shift to IL-17-producing FOXP3^+^ T cells might induce the expression of LAG3. Moreover, Egr2, which is a key transcription factor of CD25^−^LAG3^+^ T cells (50), is reported to be a positive regulator for Th17 cell development in a network analysis (51). Egr2 might be associated with the expression of LAG3 in IL-17-producing FOXP3^+^ T cells in SLE. However, the relationship between LAG3 and RORC or IL-17 production remains to be elucidated.

There are several limitations in our analysis. First, the number of patients (26 SLE patients, 30 RA patients and 30 healthy controls) were relatively small. Second, because the number of patients performing cell frequencies analysis of before and after treatment was limited, further confirmation is necessary regarding decrease of frequencies of CD25^+^LAG3^+^ T cells after treatment. Third, there is a possibility that anti-human LAG3 antibody might affect the result of T cell suppression assay. Forth, since the number of IL-17^+^ cells in CD4^+^CD25^+^LAG3^+^ T cells is not so high, there might be another mechanism for CD4^+^CD25^+^LAG3+ T cells not to show evident suppressive activity.

In summary, CD25^+^LAG3^+^ T cells are low in frequency in HC and RA, but significantly greater in SLE. The frequency of CD25^+^LAG3^+^ T cells was significantly positively correlated with the SLEDAI score and was reduced by treatment. CD25^+^LAG3^+^ T cells expressed both FOXP3, RORC and IL-17A, suggesting that they were IL-17-producing FOXP3^+^ T cells.

## Data Availability

The datasets generated for this study are available on request to the corresponding author.

## Ethics Statement

All clinical investigations conformed to the Declaration of Helsinki principles and were approved by the ethics committee of the University of Tokyo (No. 10154 and G3582). Peripheral blood and clinical data were collected after getting written informed consent in accordance with our ethical review board.

## Author Contributions

RK and SS equally contributed to this manuscript. RK, SS, YT, HT, SN, TO, KY, and KF conceived, designed and analyzed the experiments, and contributed to writing the manuscript. RK and SS carried out all experiments. KS, NH, YN, KK, ST, and HK helped with obtaining human samples and clinical data.

### Conflict of Interest Statement

SS received financial support or fees from AbbVie, Eisai, Chugai, UCB, BMS, Takeda, AstraZeneca, Pfizer, MitsubishiTanabe, Novartis Pharma, and Asahi Kasei. TO received financial support or fees from Chugai, Novartis Pharma. KY received financial support or fees from AbbVie, Astellas, BMS, Daiichi-Sankyo, MitsubishiTanabe, Pfizer, Sanofi, Santen, Takeda, Teijin, Boehringer Ingelheim, Chugai, Eisai, Ono, Taisho Toyama, UCB, ImmunoFuture, Asahi Kasei, and Janssen. KF received financial support or fees from Astellas, BMS, Daiichi-Sankyo, MitsubishiTanabe, Pfizer, Santen, Takeda, Chugai, Eisai, Taisho Toyama, UCB, and Janssen. The remaining authors declare that the research was conducted in the absence of any commercial or financial relationships that could be construed as a potential conflict of interest.
